# Recombinant protein susceptibility to proteolysis in the plant cell secretory pathway is pH‐dependent

**DOI:** 10.1111/pbi.12928

**Published:** 2018-05-02

**Authors:** Philippe V. Jutras, Marie‐Claire Goulet, Pierre‐Olivier Lavoie, Marc‐André D'Aoust, Frank Sainsbury, Dominique Michaud

**Affiliations:** ^1^ Centre de recherche et d'innovation sur les végétaux Université Laval Quebec City QC Canada; ^2^ Medicago Inc. Quebec City QC Canada; ^3^ Australian Institute for Bioengineering and Nanotechnology The University of Queensland St Lucia Qld Australia

**Keywords:** cleavable peptide linkers, endogenous proteolysis, fusion protein design, influenza virus M2 proton channel, leaf cell secretory pathway, *Nicotiana benthamiana*

## Abstract

Cellular engineering approaches have been proposed to mitigate unintended proteolysis in plant protein biofactories, involving the design of protease activity‐depleted environments by gene silencing or *in situ* inactivation with accessory protease inhibitors. Here, we assessed the impact of influenza virus M2 proton channel on host protease activities and recombinant protein processing in the cell secretory pathway of *Nicotiana benthamiana* leaves. Transient co‐expression assays with M2 and GFP variant pHluorin were first conducted to illustrate the potential of proton export from the Golgi lumen to promote recombinant protein yield. A fusion protein‐based system involving protease‐sensitive peptide linkers to attach inactive variants of tomato cystatin SlCYS8 was then designed to relate the effects of M2 on protein levels with altered protease activities *in situ*. Secreted versions of the cystatin fusions transiently expressed in leaf tissue showed variable ‘fusion to free cystatin’ cleavage ratios, in line with the occurrence of protease forms differentially active against the peptide linkers in the secretory pathway. Variable ratios were also observed for the fusions co‐expressed with M2, but the extent of fusion cleavage was changed for several fusions, positively or negatively, as a result of pH increase in the Golgi. These data indicating a remodelling of endogenous protease activities upon M2 expression confirm that the stability of recombinant proteins in the plant cell secretory pathway is pH‐dependent. They suggest, in practice, the potential of M2 proton channel to modulate the stability of protease‐susceptible secreted proteins *in planta* via a pH‐related, indirect effect on host resident proteases.

## Introduction

The value of plants as biological factories for medically useful proteins has been confirmed in recent years with the approval of a first plant‐made biopharmaceutical for human therapy, the successful production of therapeutic antibodies in plants during the recent Ebola virus outbreak and the increasing number of plant‐derived proteins subject to clinical trials for human use (Lomonossoff and D'Aoust, 2016; Ma *et al*., 2015; Sack *et al*., 2015). Significant progress has been made over the past 15 years to optimize transgene expression in plant systems and to develop strategies for protein production and recovery from various plant sources (Makhzoum *et al*., 2014; Streatfield, 2007). Progress has also been made to dissect the particularities of protein biosynthesis and posttranslational maturation in plant cells, helpful to ensure proper maturation of the expressed proteins and to implement new cellular functions for protein engineering *in planta* (Faye *et al*., 2005; Gomord *et al*., 2010; Mandal *et al*., 2016). A key challenge at present to further bolster the use of plants as protein biofactories is to maximize the overall quality and yield of the resulting product. A variety of useful proteins have now been produced in plant systems (Fahad *et al*., 2015), but the successful expression of several others remains challenging. Proteins face biochemical and physicochemical constraints in plant cell environments, such as the occurrence of proteases or the establishment of pH gradients between cell compartments, that may strongly impair their accumulation by negative effects on their structural properties (Benchabane *et al*., 2008).

Unintended proteolysis is a key factor influencing the quality and steady‐state levels of foreign proteins in plants, notably in the cell secretory pathway (Doran, 2006; Mandal *et al*., 2016; Pillay *et al*., 2014). Proteolytic enzymes are implicated in many important physiological and developmental processes in plants (Schaller, 2004; van der Hoorn, 2008), but their abundance and diversity in most tissues is a burden to the effective production of several proteins (Benchabane *et al*., 2008; Goulet *et al*., 2012). Protein engineering strategies have been proposed to elude unintended proteolytic degradation, involving the removal of protease‐susceptible sites by site‐directed mutagenesis or protein domain substitution (Hehle *et al*., 2016; Zischewski *et al*., 2015), peptide sorting signals for protein accumulation in alternative cellular compartments (Faye *et al*., 2005; Harrison *et al*., 2011) or stabilizing fusion partners for protein sequestration in protein bodies (Conley *et al*., 2011; Torrent *et al*., 2009). Cellular engineering strategies have been also proposed to reduce proteolytic activities *in situ*, involving protease gene silencing with antisense DNA or RNAi sequences (Duwadi *et al*., 2015; Mandal *et al*., 2014), or the co‐expression of accessory protease inhibitors to generate protease activity‐depleted environments (Goulet *et al*., 2012; Jutras *et al*., 2016; Robert *et al*., 2013).

In this study, we assessed the impact of influenza virus M2 proton channel (Holsinger *et al*., 1994) on endogenous protease activities and recombinant protein processing in the leaf cell secretory pathway of *Nicotiana benthamiana* used as an expression host. M2 forms tetrameric transmembrane proton channels in the secretory pathway of infected mammalian cells, helpful to maintain a high pH favourable to viral protein folding and stability in the trans‐Golgi network (Cady *et al*., 2009). Transient expression of this transport protein in *N. benthamiana* leaves was shown recently to trigger a similar pH increase in the Golgi compartments, useful in practice to stabilize the structural conformation of pH‐labile recombinant proteins and peptides (Jutras *et al*., 2015). Here we aimed at assessing whether M2 also exerts an indirect stabilizing effect on recombinant proteins, including acid‐tolerant proteins, via an alteration of host protease activities upon proton export. The hydrolytic activity of broad‐spectrum cysteine (Müntz and Shutov, 2002; Richau *et al*., 2012), aspartate (Simões and Faro, 2004) and serine (Antão and Malcata, 2005) proteases such as those found in the secretory pathway of plant cells (Goulet *et al*., 2012) is generally pH‐dependent and could in theory be influenced, positively or negatively, in Golgi compartments presenting an altered pH. We addressed this question with *N. benthamiana* as an expression host and biologically inactive protein fusions integrating peptide linkers differentially susceptible to proteolytic degradation.

## Results and discussion

### M2 proton channel co‐expression promotes the accumulation of GFP variant pHluorin in leaves

**Figure 1:**
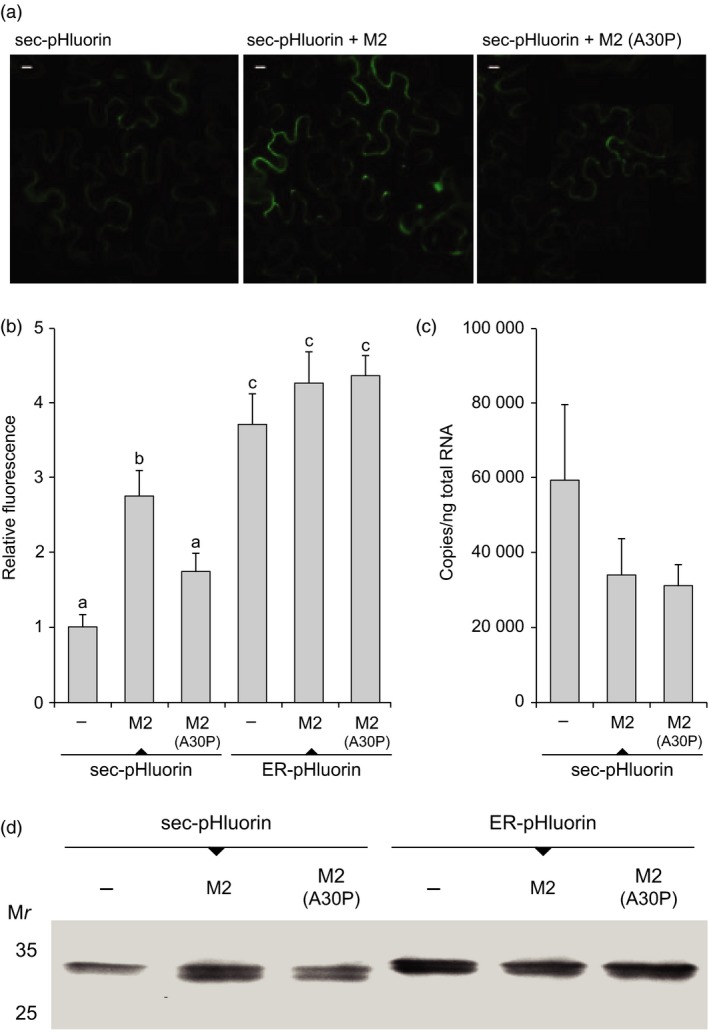
Recombinant pHluorin expression in the secretory pathway of *N. benthamiana* leaf cells. The reporter protein was transiently expressed in agroinfiltrated leaves, alone (−), along with M2 channel or along with A30P, a single mutant of M2 unable to drive proton transport. (a) Confocal microscopy detection of fluorescence in pHluorin‐transfected leaf cells. Image data were acquired under excitation and emission wavelengths of 488 nm and 515 nm, respectively. Bars = 10 μm. (b) Fluorescence emission at 515 nm in transfected leaf protein extracts. Data are presented relative to pHluorin expressed alone (value of 1). Each value is the mean of three biological (plant) replicates ± SE. (c) mRNA transcripts for pHluorin in pHluorin‐transfected leaves. Each value is the mean of five biological (plant) replicates ± SE. (d) Immunodetection of pHluorin in leaf protein extracts. A same volume of extract was loaded in each well, corresponding to approximately 12 μg of protein. Numbers on the left indicate the position of molecular weight markers. All experiments on this figure were conducted with leaves of the same physiological age collected 6 d postinfiltration. Bars with different letters on panel b are significantly different (post‐anova LSD; *P < *0.05). sec‐pHluorin, pHluorin secreted in the apoplast; ER‐pHluorin, pHluorin retained in the ER.

Agroinfiltrations were first conducted to assess the impact of M2 channel co‐expression on the accumulation of reporter protein pHluorin in leaves (Figure 1). This functional variant of GFP is structurally stable over a range of pH (Miesenböck *et al*., 1998; Schulte *et al*., 2006) encompassing those neutral to mildly acidic pH conditions characteristic of the cell secretory pathway (Paroutis *et al*., 2004; Figure S1). As expected, fluorescence was readily detected in protein extracts of leaves transfected to express pHluorin, compared to protein extracts of mock‐infiltrated leaves only showing chloroplastic autofluorescence background (not shown). Fluorescence was also detected in leaves infiltrated to co‐express pHluorin and M2, at a relative intensity two to three times the intensity measured in leaves expressing pHluorin alone (post‐anova LSD, *P < *0.05; Figure 1a,b). This promoting effect of M2 on pHluorin content was not observed with A30P (post‐anova LSD, *P > *0.05), an inactive mutant of M2 properly expressed in *N. benthamiana* leaves (Jutras *et al*., 2015) but unable to drive proton transport out of the secretory pathway (Holsinger *et al*., 1994). A post‐transcriptional, proton transport‐related effect of M2 on pHluorin content was further suggested by real‐time RT‐PCR assays that showed no increase in transcript numbers for pHluorin in M2‐transfected leaves (anova;* P > *0.05; Figure 1c).

We showed previously the pH‐altering effect of M2‐induced proton transport in *N. benthamiana* leaf cells to be Golgi‐specific, downstream of the endoplasmic reticulum (ER) complex (Jutras *et al*., 2015). We here fused a Lys‐Asp‐Glu‐Leu (KDEL) ER‐retention peptide at the C terminus of pHluorin to assess whether the increased accumulation of this protein in leaves was also the result of a post‐ER effect. In accordance with fluorescence emission data above, secreted pHluorin content as visualized on immunoblots was greater in leaf tissue also expressing M2 (*see* sec‐pHluorin samples on Figure 1d). By contrast, M2 co‐expression had no impact on the content of ER‐retained pHluorin (ER‐pHluorin samples on Figure 1d), then confirming a post‐ER effect of proton export on pHluorin accumulation in the cell secretory pathway. M2 channel activity leading to pH increase was shown previously to promote the stability of acid‐labile recombinant proteins and peptides in the Golgi network of *N. benthamiana* leaf cells (Jutras *et al*., 2015). We now propose a complementary, indirect effect of M2 *in planta,* by which proton transport out of the Golgi lumen would also influence the stability and yield of acid‐tolerant recombinant proteins.

### A fusion protein system for peptide cleavage monitoring in the cell secretory pathway

**Figure 2:**
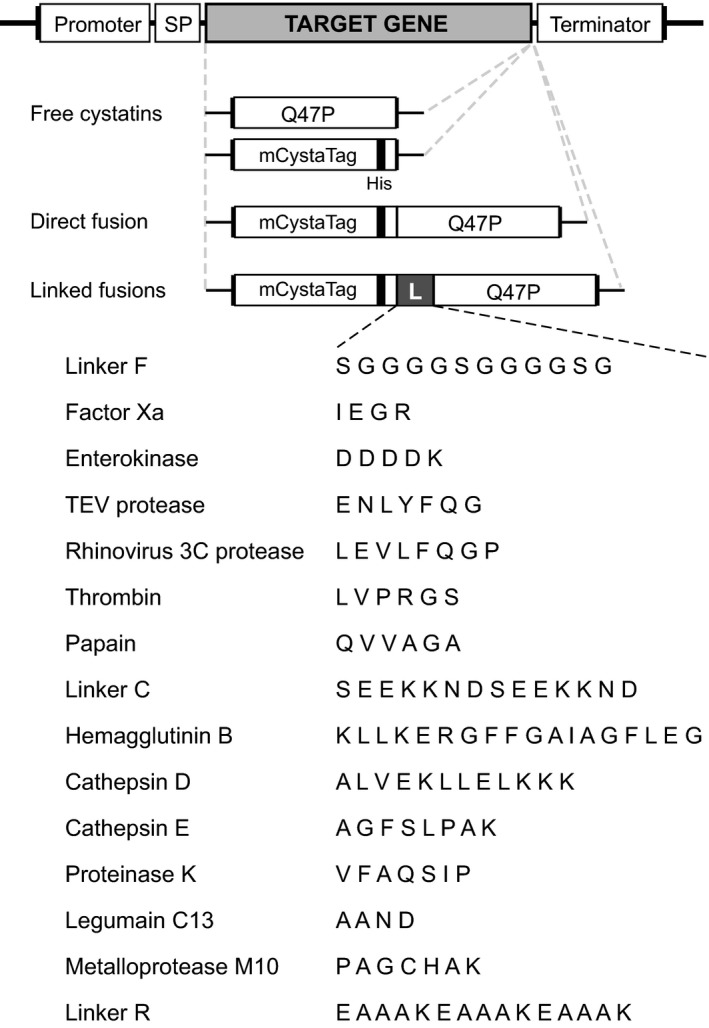
mCystaTag–Q47P fusions and peptide linkers designed for the experiments. Two single variants of tomato cystatin SlCYS8, Q47P and mCystaTag, were used as inactive partner moieties for the fusion constructs (*see* main text for details). Peptide sequences susceptible to different proteases were used as linkers (L) to attach the protein partners. Constructs were also produced for the free fusion partners expressed alone and for a no‐linker, ‘direct’ fusion used as control for stability assessment assays. All expressed proteins harboured an N‐terminal signal peptide (SP) for cellular secretion. Assembled coding sequences (target gene) were inserted in a pCambia 2300 expression vector, between a duplicated Cauliflower mosaic virus 35S promoter sequence (Promoter) in 5′ and a nopaline synthase terminator sequence (Terminator) in 3′. Linker sequences were named according to Sainsbury *et al*. (2013) (Linkers F, C and R) or based on model proteases reported to cleave the corresponding amino acid string (*see* Table 1).

Several studies have described the negative effects of host resident proteases on the stability of secreted proteins in plant expression systems (Benchabane *et al*., 2008; Doran, 2006). Several studies have also described strategies to alleviate this problem, notably involving the silenced expression of endogenous protease targets (Duwadi *et al*., 2015; Mandal *et al*., 2014) or the co‐expression of accessory protease inhibitors to prevent unintended proteolysis *in situ* (Jutras *et al*., 2016; Robert *et al*., 2016). We here assessed whether the post‐ER‐positive effect of M2 channel on secreted protein (e.g. pHluorin) content could also be associated with an alteration of host protease activities, keeping in mind the influence of pH on the hydrolytic efficiency of most proteases (Barrett *et al*., 1998). Protease‐labile translational fusion proteins were designed to address this question, all of them including two inactive variants of tomato cystatin SlCYS8 (Kiggundu *et al*., 2006) attached together with peptide linkers differentially susceptible to Cys, Ser, Asp or metal‐dependent proteases (Figure 2, Table 1). Q47P (or ^Q47P^SlCYS8), a single mutant of SlCYS8 bearing a proline in place of the original glutamine at position 47 (Sainsbury *et al*., 2012a), was used as a biologically inert C‐terminal protein partner for the fusions. A poly‐His‐tagged analogue of Q47P, referred to as mCystaTag (or ^Q47P^ *Cysta*Tag, for mutated CystaTag) in reference to the recently described SlCYS8 variant CystaTag (Sainsbury *et al*., 2016), was used as an N‐terminal protein partner.

**Table 1:** Expected susceptibility of selected peptide linkers to proteases of different functional families

Protease family	Peptide linker	References
Cysteine proteases	Legumain C13	Smith *et al*. (2014)
	Papain	Gauthier and Moreau (1993)
	Linker F	Sainsbury *et al*. (2013)
	TEV protease	Sainsbury *et al*. (2016)
	Human Rhinovirus 3C	Sainsbury *et al*. (2016)
Serine proteases	Thrombin	Terpe (2003)
	Factor Xa	Sainsbury *et al*. (2016)
	Enterokinase	Sainsbury *et al*. (2016)
	Linker C	Horn *et al*. (2005)
	Proteinase K	Horn *et al*. (2005)
Aspartic proteases	Cathepsin D	Sun *et al*. (2013)
	Cathepsin E	Abd‐Elgaliel and Tung (2010)
Metal‐dependent proteases	Metalloprotease M10	Mandal *et al*. (2010)
Uncharacterized	Hemagglutinin B	Wang *et al*. (2008)
	Linker R (acid‐labile)	Jutras *et al*. (2015)

**Figure 3:**
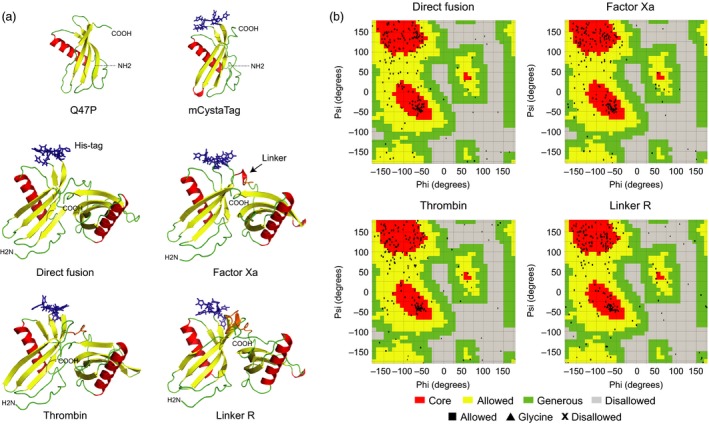
*In silico* modelling of the mCystaTag–Q47P fusions. (a) 3D models for Q47P, mCystaTag and four representative mCystaTag–Q47P fusions as inferred using the I‐TASSER server for protein structure prediction (Roy *et al*., 2010). Models were built with the NMR structure of rice cystatin I (PDB 1EQK) as a template. Peptide linkers are shown in orange (*see* arrow), the poly‐His motif of mCystaTag in blue. (b) Ramachandran plots for stereochemical quality assessment of the fusion structure models shown on panel a. Most amino acid residues (black dots) fell within the ‘core’ (red) and ‘allowed’ (yellow) areas of the plots, as expected for good quality structural models (Morris *et al*., 1992). Four models are shown on panel a; models for the other 12 fusions are shown in Figure S2.

Peptide linkers are known to influence the overall stability and accumulation rate of hybrid fusions in heterologous protein expression systems (Sainsbury *et al*., 2012b; Wriggers *et al*., 2005), including *N. benthamiana* (Sainsbury *et al*., 2013). Structural models were here produced *in silico* for the mCystaTag–Q47P fusions to confirm their structural robustness and the steric accessibility of their peptide linkers to host plant proteases (Figure 3). Amino acid sequences for the fusion hybrids were submitted to the Iterative Threading Assembly Refinement (I‐TASSER) server for protein structure prediction (zhanglab.ccmb.med.umich.edu/i‐tasser; Roy *et al*., 2010) to generate tertiary structure simulations and to select, among possible models, the best model for each fusion based on the ‘lowest free energy conformation’ criterium (Figures 3a and S2). Ramachandran plots were then produced online through the VADAR web server (Willard *et al*., 2003) to assess the stereochemical quality of the selected models (Laskowski *et al*., 1993; *see* Figure 3b for some examples). For all fusions, most amino acids fell within the ‘core’ (red) and ‘allowed’ (yellow) areas of the Ramachandran plots (black dots on Figure 3b), thus supporting the validity of our structure predictions (Morris *et al*., 1992).

A visual assessment of the models suggested very low steric interference between the two fusion partners whatever the fusion considered, including the head‐to‐tail, linker‐free ‘direct fusion’ (Figures 3a and S2). All models exhibited a two‐domain tertiary structure closely matching, on each side of the hybrid, the predicted structures of SlCYS8 or its CystaTag variant (Sainsbury *et al*., 2016). As expected given the intrinsic structural robustness of plant cystatins (Benchabane *et al*., 2010) and the reported ability of some of them to act as multidomain fusion inhibitors *in vivo* (Kouzuma *et al*., 2000; Nissen *et al*., 2009), the linkers had little effect on the predicted structure of either cystatin partners. Despite variation in size, length and physicochemical properties, the linkers also had little influence on the distance between, and relative orientation of, the two cystatin moieties. This was even the case with Linker R ([EAAAK]_3_; Figure 3a), a rigid linker often used in fusion protein design to maintain a minimal distance and to prevent interdomain interactions between noncognate fusion partners (Arai *et al*., 2001). These *in silico* inferences, together with favourable aliphatic index (Ikai, 1980) and instability index (Guruprasad *et al*., 1990) values calculated using the Protparam tool for protein analysis (Gasteiger *et al*., 2005; Table S1), supported overall the thermodynamic feasibility of expressing the 16 cystatin fusions under a stable form in heterologous cellular environments. Our models also suggested an adequate steric accessibility of their peptide linkers and hence their potential usefulness to monitor host‐mediated proteolytic events in the cell secretory pathway.

### The mCystaTag–Q47P fusions are differentially susceptible to proteolytic cleavage *in planta*

**Figure 4:**
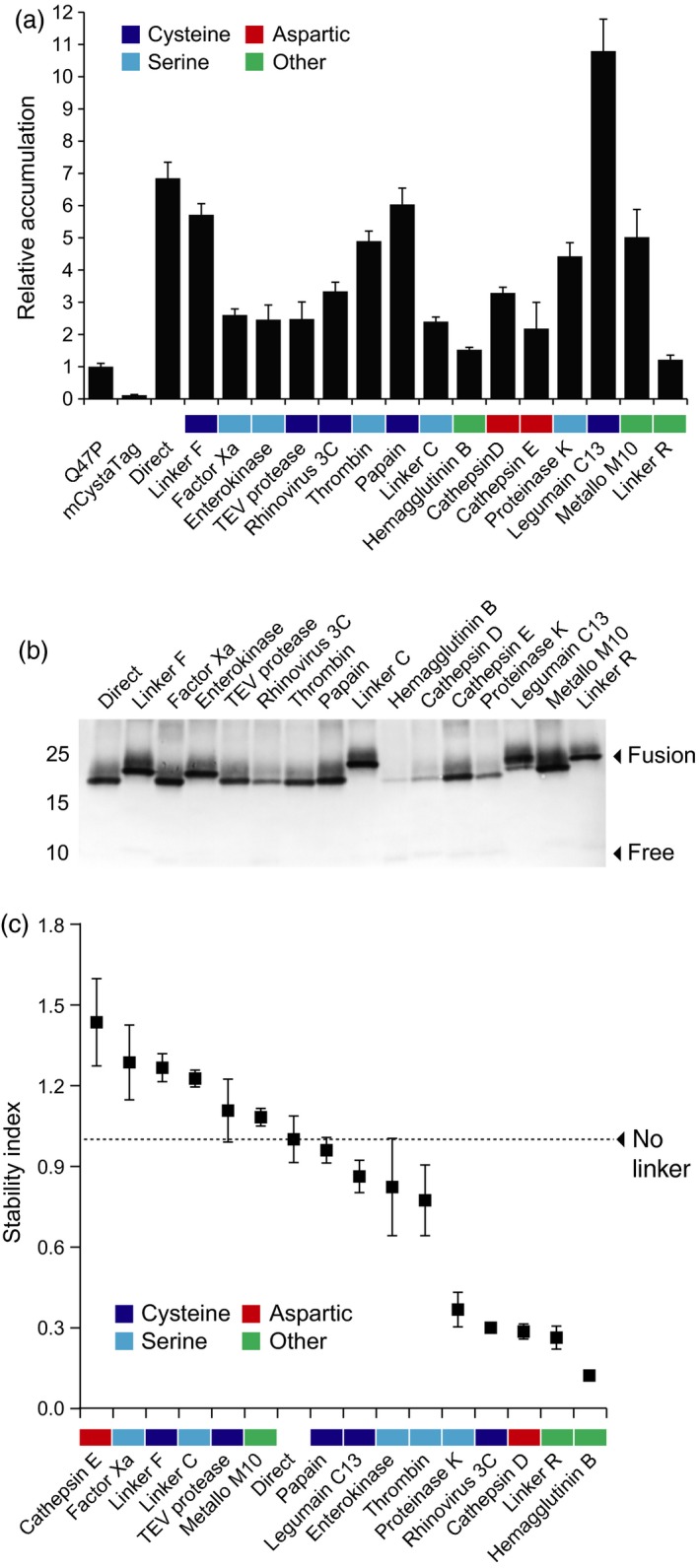
Expression and stability of the mCystaTag–Q47P hybrids in *N. benthamiana* leaves. (a) ELISA‐assayed cystatin content in leaves on a soluble protein‐specific basis. Data are expressed compared to free, single‐domain Q47P (assigned an arbitrary value of 1). (b) Immunodetection of the mCystaTag–Q47P fusion hybrids in ELISA‐assayed samples containing a same amount of antibody‐reacting cystatin domains. Numbers on the left indicate the position of molecular weight markers. (c) A ‘stability index’ scale for the mCystaTag–Q47P fusions, as determined by densitometric analysis of fusion protein signals detected on nonsaturated immunoblots. Index values were calculated relative to an arbitrary value of 1.0 assigned to the no‐linker, direct fusion (dotted line on graph). Colours on panels a and c were assigned to the linkers based on their assumed susceptibility to specific protease families (*see* Table 1). Data on these two panels are the mean of three independent (plant) replicate values ± SE.

An array of Asp, Cys, Ser and metal‐dependent proteases are found in the plant cell secretory pathway, which together represent a burden to the effective production of several heterologous proteins (Doran, 2006; Goulet *et al*., 2012). Leaf transfection assays were here conducted with M2 proton channel and the mCystaTag–Q47P fusions to formally document the negative effects of these hydrolytic enzymes on recombinant protein integrity in the secretory pathway and to confirm the influence of an alleviated pH gradient on these effects as discussed above for pHluorin co‐expressed with M2 (*see* Figure 1). The cystatin fusions were first expressed in the absence of M2 to measure their relative accumulation rate in leaf tissue, compared to single‐domain Q47P and mCystaTag used as fusion partner‐free controls. As visualized following SDS‐PAGE and as expected given the structure inferences above, a protein band of 23–25 kDa was detected in all leaf extracts expected to contain a cystatin fusion, albeit at varying levels (not shown). Quantitative enzyme‐linked immunosorbent assays (ELISA) were performed with anti‐SlCYS8 polyclonal antibodies (Robert *et al*., 2013) to compare the relative amounts of ‘cystatin domains’ in leaf extracts (Figure 4). Similar to Sainsbury *et al*. (2013) reporting a strong stabilizing effect for SlCYS8 used as a fusion partner to human serpin α_1_‐antichymotrypsin, 14 fusions, of the 16 assessed, showed a cystatin domain content two to several times the content measured in leaves transfected to express single‐domain Q47P or mCystaTag (Figure 4a).

These data clearly pointed to the high conformational stability of Q47P and mCystaTag as basic components of the cystatin fusions, and hence to the eventual significance of the selected linker peptides as key determinants for the stability of cystatin hybrids *in planta*. Immunoblot analyses were conducted to address this question, using fixed amounts of cystatin domains and assuming a link between protein signal intensities in the 23–25 kDa range on nitrocellulose sheets and the abundance of intact (unprocessed) fusions in leaf extracts (Figure 4b). Signals of variable intensity were detected depending on the linker, ranging from strong for some fusions to very faint for some others. For instance, a strong signal was observed for the Linker F fusion, as reported previously for an SlCYS8–α_1_‐antichymotrypsin fusion harbouring the same linker (Sainsbury *et al*., 2013). By contrast, a very faint signal was observed for the influenza hemagglutinin B (HAB) fusion, in agreement with the reported protease lability of this viral peptide sequence in the secretory pathway of eukaryotic cells (Wang *et al*., 2008). The immunodetected fusions were quantified by densitometry to calculate a ‘stability index’ for the 15 linkers, defined as the mean signal intensity of their corresponding fusion at 23–25 kDa relative to the mean signal intensity of linker‐free, direct fusion hybrid (assigned a stability index of 1.0; Figure 4c). A range of stability indices were calculated, ranging from 1.2 to more than 1.4 for stable linkers such as Linker F, Linker C or the peptide cleavage site of human factor X_a_, to 0.3 or <0.3 for labile linkers such as the HAB viral sequence or the peptide cleavage sites of cathepsin D and human rhinovirus 3C protease. No obvious link could be established between the stability index of the linkers and their assumed susceptibility to specific classes of proteases (*see* colour code for Cys, Ser and Asp proteases on Figure 4a,c). Likewise, no formal link could be established between stability indices, length or charge of the linkers (Figure 2), and/or steric stability of the fusions as inferred *in silico* (Figure 3, Table S1). These data highlighting the complexity and specificity of secreted protease patterns in plant leaf cells underlined overall the challenges still to be met in practice for a rational control of secreted protein processing *in planta*. They pointed, on the other hand, to the eventual usefulness of our mCystaTag–Q47P fusions as a tool to detect alterations of resident protease activity patterns in the cell secretory pathway.

### Proteolytic processing of the mCystaTag–Q47P fusions is pH‐dependent

**Figure 5:**
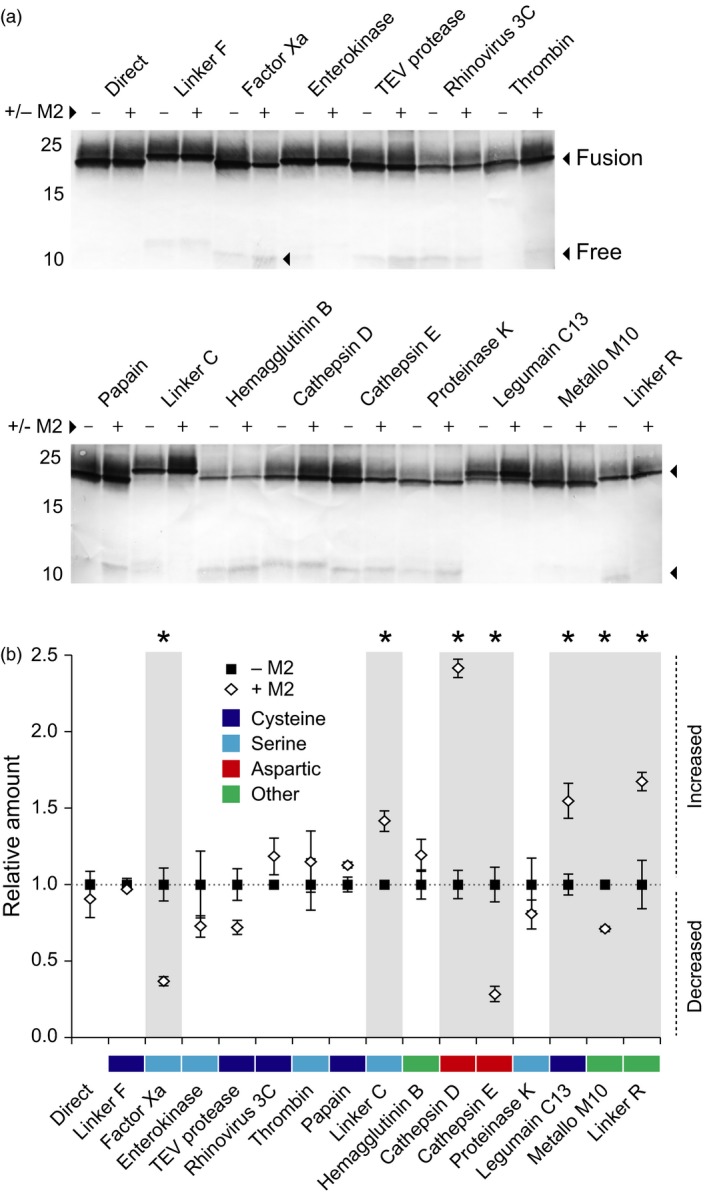
Stability of the mCystaTag–Q47P hybrids expressed alone (−) or along with M2 (+) in *N. benthamiana* leaves. (a) Immunodetection of mCystaTag–Q47P fusions in ELISA‐assayed samples containing a same amount of antibody‐reactive cystatin domains. Numbers on the left indicate the position of molecular weight markers. (b) Relative amount of each fusion expressed alone or along with M2, as determined by densitometric analysis of fusion protein signals detected on non‐saturated immunoblots. Data for the fusions co‐transfected with M2 (white diamonds) are expressed relative to the signals detected in protein extracts of leaves transfected with the corresponding fusion construct only (black squares; relative value of 1.0). Colours on panel b were assigned to the linkers based on their assumed susceptibility to specific protease families (*see* Table 1). Data on this panel are the mean of three independent (plant) replicate values ± SE. Asterisks indicate a statistically different value in the presence of M2 (Student's *t*‐test, *P < *0.05).

**Figure 6:**
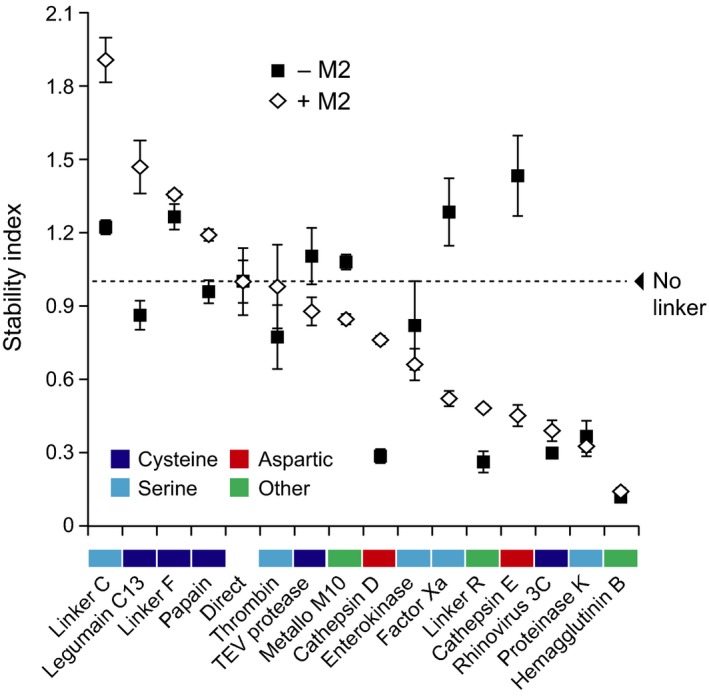
A comparison of stability index values for the mCystaTag–Q47P fusion hybrids expressed alone or along with M2 in *N. benthamiana* leaves. The stability indices were determined by densitometric analysis of fusion protein signals detected on non‐saturated immunoblots. White diamonds refer to the fusions co‐expressed with M2, black squares to the fusions expressed alone (*see* Figure 4c). All index values were calculated relative to an arbitrary value of 1.0 assigned to the no‐linker, direct fusion (dotted line). Colours on the *x*‐axis were assigned to the linkers based on their assumed susceptibility to specific protease families (*see* Table 1). Each datum on this figure is the mean of three independent (plant) replicate values ± SE.

We here used the stability index ranking determined above for the mCystaTag–Q47P fusions (Figure 4c) as a reference to confirm the impact of M2 channel co‐expression on resident protease activities and recombinant protein stability *in planta* (Figures 5 and 6). Most protease activities in biological systems are influenced by the pH, but preferences for specific ranges of this variable depend on the protease or protease family (Barrett *et al*., 1998). Accordingly, seven cystatin fusions of the 16 assessed had their integrity and resulting content in leaf tissue affected when co‐expressed with M2 (Student's *t*‐tests; *P < *0.05), unlike the nine others showing unchanged levels (Figure 5a). Of the seven fusions influenced by M2 co‐expression, four were found at higher levels in leaf extracts while the three others showed reduced contents (Figure 5b). Cystatin fusions with the peptide cleavage sequences of cathepsin D and C13 legumains were found at higher levels in M2‐expressing leaves, in line with the preference of these proteases for acidic environments (Dall and Brandstetter, 2012; Sun *et al*., 2013). The Linker R fusion was also stabilized – and its free cystatin domain content reduced (Figure S3) – in M2‐expressing leaves, consistent with the autoproteolytic activity of this peptide at low pH (Wu *et al*., 2009; Zhao *et al*., 2008) and its recently reported stabilization in *N. benthamiana* leaves expressing M2 (Jutras *et al*., 2015). By contrast, cystatin fusions with the cleavage sequences of human factor X_a_ and cathepsin E showed reduced levels – and their released free domains increased levels (Figure S3) – in M2‐expressing leaves. *In vitro* incubation assays with immobilized metal affinity chromatography (IMAC)‐purified protein preparations confirmed the intrinsic stability of these two fusions in neutral to mildly acidic pH environments (Figure S4), thus ruling out the possibility of nonenzymatic cleavage and supporting the hypothesis of altered proteolytic patterns affecting recombinant protein integrity in the cell secretory pathway.

Immunoblot analyses were conducted with fixed amounts of ELISA‐assayed cystatin domains to generate a relative stability index ranking for the peptide linkers co‐expressed with M2 (Figure 6). Protein signals of variable intensity were detected for the 16 fusions, from strong to very faint as observed above for the fusions expressed alone. Some fusions showing a strong signal in the absence of M2 still showed a strong signal when co‐expressed with this protein, while some others showing a faint signal as expressed alone still showed a faint signal in M2‐expressing leaves. By comparison, a number of fusions showed a strongly altered stability index in M2‐expressing leaves, associated with an increased or decreased content in leaf extracts. Linker C, with a relative stability index of 1.2 in the absence of M2, showed for instance a stability index of more than 1.8 in M2‐expressing leaves. In sharp contrast, cathepsin E linker showed a stability index of about 0.4 in M2‐expressing leaves, approximately 1.0 unit lower than the stability index determined for the same fusion expressed alone. Together, these data pointed to a significant remodelling of the endogenous protease activity complement upon M2 channel expression in *N. benthamiana* leaves and confirmed that the stability of recombinant proteins in the plant cell secretory pathway is pH‐dependent. These data suggested, in practice, the potential of proton channel expression to modulate the stability of protease‐susceptible secreted proteins and peptides *in planta* via an indirect, pH‐related effect on host resident proteases.

## Conclusion

Our goal in this study was to assess the impact of influenza virus M2 proton channel on endogenous protease activity patterns and recombinant protein processing in the cell secretory pathway of *N. benthamiana* leaves. Transient co‐expression assays with M2 and reporter protein pHluorin first allowed to illustrate the potential of proton export in promoting the accumulation of a recombinant protein in the cell secretory pathway. A fusion protein system involving inactive variants of SlCYS8 fused in tandem with peptide linkers differentially susceptible to proteolysis was then developed, which allowed to relate the effects of M2 on recombinant protein levels to an alteration of host protease activities upon proton export. M2 channel transient expression in *N. benthamiana* leaves was shown recently to trigger a notable pH increase in the Golgi lumen, useful to stabilize pH‐labile recombinant proteins and peptides in the cell secretory pathway (Jutras *et al*., 2015). We here report a complementary effect of the viral channel, by which proton transport out of the Golgi influences the integrity of acid‐tolerant recombinant proteins via an indirect, pH‐related effect on resident protease activities. Work is underway to detect eventual synergistic effects between this new way of modulating endogenous protease activity patterns and previously described strategies for the ectopic inhibition of specific protease family targets (Jutras *et al*., 2016; Robert *et al*., 2016). Research efforts will be welcome in coming years to evaluate the feasibility of combining the mCystaTag affinity handle, M2 channel co‐expression and protease‐stable linkers such as Linker F or Linker C to improve the yield of recombinant proteins *in planta* and facilitate their purification following extraction from leaf tissue. Research efforts will also be welcome to characterize the effects of M2 expression at the proteome scale, taking into account the demonstrated importance of pH homeostasis for proper protein maturation and trafficking in the cell secretory pathway (Bassil *et al*., 2012; Pittman, 2012) and the cell‐wide ‘off‐target’ impacts of ectopic protease inhibition on the leaf proteome (Badri *et al*., 2009; Goulet *et al*., 2010).

## Experimental procedures

### Fusion protein design and *in silico* protein modelling

Peptide linkers to design the mCystaTag–Q47P fusions were chosen based on their reported susceptibility to specific proteases (Figure 2; Table 1). Three‐dimensional structural models for the fusions were inferred *in silico* using the I‐TASSER suite for protein structure and function prediction (Yang *et al*., 2015). Ramachandran plots to validate the stereochemical quality of the models (Willard *et al*., 2003) were produced using the online program VADAR for structural analysis of protein coordinate data (http://vadar.wishartlab.com). Aliphatic and instability indices for the fusion sequences were calculated using the EXPASY server ProtParam tool (http://web.expasy.org/protparam).

### Plasmid constructs

Gene constructs for pHluorin, M2, M2 inactive variant A30P, Q47P and its CystaTag variant were described previously (Jutras *et al*., 2015; Sainsbury *et al*., 2013, 2016). Gene constructs for the mCystaTag–Q47P fusions were produced using a synthetic DNA string (ATUM, Newark, CA) encoding native SlCYS8 (GenBank Accession No. AF198390) fused at the C‐terminal end of the CystaTag (Sainsbury *et al*., 2016). Glutamine to proline point mutations was introduced at position 47 of both fusion partners by the Quickchange mutagenesis method (Agilent Technologies, Mississauga ON, Canada) to produce a ‘Q47P’ inactive version of the fusion template (Sainsbury *et al*., 2013). Peptide linker sequences were then inserted between the two fusion partners by routine PCR procedures, for a total of 16 translational fusions including a ‘direct’, linker‐free fusion control. All expressed proteins included a native [M2 and A30P variant] (GenBank Accession HQ008884) or heterologous [pHluorin, Q47P, CystaTag and mCystaTag–Q47P fusions] (Shorrosh and Dixon, 1991) N‐terminal signal peptide for co‐translational inclusion in the cell secretory pathway. Transgenes were assembled in a pCambia 2300 expression vector harbouring a gene expression cassette for the silencing suppressor protein p19 (CAMBIA, Canberra, ACT, Australia), between a duplicated Cauliflower mosaic virus (CaMV) 35S promoter in 5′ position and a nopaline synthase (NOS) terminator sequence in 3′ position. All gene constructs were proofchecked by automatic DNA sequencing. The pCambia vectors were maintained in *Agrobacterium tumefaciens*, strain AGL1 (Lazo *et al*., 1991), until use for the agroinfiltrations.

### Transient expression in *Nicotiana benthamiana*

Bacterial cultures for leaf agroinfiltration were grown to stable phase in Luria‐Bertani medium supplemented with appropriate antibiotics and then harvested by gentle centrifugation at 4000 *g* for 5 min at 20 °C. The bacterial pellets were resuspended in 10 mm MES (2‐[N‐morpholino]ethane sulphonic acid), pH 5.6, containing 10 mm MgCl_2_ to an OD_600_ of 0.5, and incubated for 2–4 h at 20 °C. For the co‐agroinfiltrations, bacterial cultures harbouring the M2 or A30P constructs were mixed at a volume ratio of 1 in 4 with bacterial cultures for pHluorin or the mCystaTag–Q47P fusions grown at the same optical density. Co‐infiltrations with bacteria harbouring a pCambia ‘empty’ (mock) vector were carried out in parallel as a control for pHluorin or SlCYS8 fusion expression in the absence of M2. Forty‐two‐day‐old *N*. *benthamiana* plants were vacuum‐agroinfiltrated with the bacterial suspension mixtures (Leuzinger *et al*., 2013), prior to plant incubation at 20 °C in a Conviron PWG36 growth chamber (Conviron, Winnipeg, MA, Canada) for heterologous protein expression. Leaf tissue for protein (or RNA) extraction and detection was harvested 6 days postinfiltration. Three to five independent replicates each including three plants were used for each treatment to minimize variation of protein (or transgene) expression levels and to allow for statistical analysis of the data.

### Protein extraction

Proteins were extracted from the main stem's third leaf (down from the apex) to ensure consistency of leaf physiological age among treatments (Robert *et al*., 2013). Infiltrated leaf tissue was harvested as leaf discs representing 160 mg of control‐infiltrated tissue and homogenized by disruption with ceramic beads (BioSpec, Bartlesville, OK) in a Mini‐Bead beater apparatus (OMNI International, Kennesaw, GQ). Total soluble proteins were extracted in three volumes of phosphate‐buffered saline (PBS), pH 7.3, containing 5 mm EDTA, 0.05% v/v Triton X‐100 and the cOmplete protease inhibitor cocktail (Roche Diagnostics, Laval, QC, Canada). Leaf lysates were clarified by centrifugation for 20 min at 20 000 *g*. Total soluble proteins were assayed according to Bradford (1976), with bovine serum albumin as a protein standard.

### pHluorin quantification

Recombinant pHluorin expression was assayed by fluorescence detection with a Fluostar Galaxy microplate reader (BMG, Offenburg, Germany) using excitation and emission filters of 485 and 515 nm, respectively. Different amounts of protein extract were diluted in 0.1 m Na_2_CO_3_, pH 10, using a mock‐infiltrated leaf control extract for primary dilutions to prevent eventual matrix effects. pHluorin levels were determined on a relative basis based on standard curves drawn from fluorescence measurements with control leaf tissue. All sample mixtures were loaded in triplicate on Costar 96‐well black polystyrene plates (Cedarlane, Burlington, ON, Canada). Measurements were made with leaf protein extracts from three independent (plant) replicates.

### RNA extraction and quantitation

Plant samples for RNA extraction were harvested as two leaf discs representing 100 mg of fresh tissue. Leaf tissue was disrupted in a Mini‐Beadbeater (OMNI International), kept on ice for 2 min, and the resulting lysates centrifuged for 20 min at 20 000 *g*. Total RNA extraction was performed using the RNeasy Plant Mini Kit (Qiagen, Mississauga ON, Canada), according to the manufacturer's instructions. RNA concentration and quality were assessed using a NanoDrop ND‐1000 spectrophotometer (NanoDrop Technologies, Wilmington, DE), before cDNA synthesis using the Omniscript Reverse Transcription Kit (Qiagen). Quantification of pHluorin transcripts was performed by real‐time RT‐PCR in 96‐well plates using the ABI PRISM 7500 Fast real‐time PCR system and custom data analysis software, version 2.0.1 (Thermo Fisher Scientific, Whaltham, MA). PCR products contained the cDNA sample preparations, SYBR Green PCR Master Mix (Thermo Fisher Scientific) and the following forward and reverse DNA primers for pHluorin transcripts: 5′‐CATTGAAGATGGAGGCGTTC‐3′ and 5′‐GAAAGGGCAGATTGTGTGTG‐3′. The relative abundance of pHluorin transcripts was determined using a DNA standard curve for the corresponding primers, produced with 2 μL of cDNA preparation following the NEB Taq polymerase routine protocol (New England Biolabs, Essex County, MA). All measurements were made with leaf samples from five independent (plant) replicates.

### Microscopy analysis

Transient expression of pHluorin in leaf tissue was visualized 6 days postinfiltration with a Nikon C1 confocal laser imaging microscope (Nikon, Melville, NY). Image data acquisition was performed using a 60× water‐immersion lens, with emission wavelength set at 515 nm and excitation scans performed at 488 nm. Control experiments were performed to account for chloroplast autofluorescence by subtracting from each image the average fluorescence background value of images acquired with uninfiltrated plants. The power and gain of each laser line were maintained at consistent levels between the experiments. Image data were analysed using the Open source software ImageJ (http://rsb.info.nih.gov/ij/), with those images at saturated intensity eliminated or set to zero using a mask. Panel A on Figure 1 shows representative images for the different treatments, each generated with at least 15 pairs of images. Images were produced with three plants, using leaf samples of the same physiological age.

### Quantitative ELISA

ELISA for the quantitation of mCystaTag–Q47P fusions were performed on Immulon 2HB ELISA plates (Immuno‐Chemistry Technologies, Bloomington, MN) coated with triplicate samples of the leaf protein extracts diluted 1:50 in PBS, pH 7.3. Nonspecific binding sites were blocked with 1% (w/v) casein in PBS, before application of rabbit anti‐SlCYS8 polyclonal antibodies (Agrisera, Vännäs, Sweden) diluted in PBS with 0.25% (w/v) casein. Goat anti‐rabbit IgG conjugated to horseradish peroxidase (Sigma‐Aldrich, Oakville ON, Canada) were used as secondary antibodies for SlCYS8–primary antibody complex detection, followed by protein signal development with the SureBlue peroxidase substrate and 3,3′,5,5′‐tetramethylbenzidine as a colour indicator (KPL, Gaithersburg, MD). Absorbance was read at 450 nm, after the addition of 1 N HCl to stop colour development. A standard curve was generated for each plate with purified SlCYS8 (Kiggundu *et al*., 2006) diluted in control extracts from empty vector‐infiltrated leaf tissue to account for eventual matrix effects. All measurements were made with leaf extracts from three independent (plant) replicates.

### Immunoblotting and protein quantitation by densitometry

pHluorin and mCystaTag–Q47P fusions were immunodetected on nitrocellulose sheets following protein separation by 12% (w/v) SDS‐PAGE in reducing conditions. pHluorin was detected with mouse anti‐GFP monoclonal antibodies (Clontech Laboratories, Mountain View, CA, diluted 1:5000) and goat anti‐mouse secondary antibodies conjugated to alkaline phosphatase (Sigma‐Aldrich, diluted 1:5000). Q47P and mCystaTag were detected with custom‐made rabbit anti‐SlCYS8 polyclonal antibodies (Robert *et al*., 2013; Agrisera, Vännäs, Sweden, diluted 1:5000) and goat anti‐rabbit secondary antibodies conjugated to alkaline phosphatase (Sigma‐Aldrich, diluted 1:5000). Nonspecific binding on nitrocellulose sheets was prevented by incubation in blocking solution (5% w/v skim milk powder in PBS, containing 0.025% v/v Tween‐20), which also served as antibody dilution buffer. Protein signals were developed with the alkaline phosphatase substrate 5‐bromo‐4‐chloro‐3‐indolyl phosphate and nitro blue tetrazolium as a colour indicator (Life Technologies, Burlington ON, Canada). Densitometric analysis of the fusion proteins was performed using the Phoretix 2D Expression software, v. 2005 (Nonlinear USA, Durham, NC) on non‐saturated immunoblot images digitalized with an Amersham Image Scanner (GE Healthcare). All measurements were made with leaf extracts from three independent (plant) replicates.

### Statistical analyses

Statistical analyses were performed using RStudio, v. 0.98.1103 (RStudio inc., Boston, MA). Analysis of variance (anova) tests were performed to compare pHluorin fluorescence rates and mRNA transcript numbers among treatments. Fischer's least significant difference (LSD) tests were then used to separate treatment means for those anova giving significant *P* values at an alpha threshold of 5%. Stability rates of the mCystaTag–Q47P hybrids expressed alone or along with M2 were compared by Student's mean comparison *t*‐tests, with an alpha threshold of 5%.

## Supporting information


**Figure S1** Intrinsic stability of GFP variant pHluorin in neutral to mildly acidic pH conditions.
**Figure S2** Complement to Figure 3: Structural models for the remaining 12 mCystaTag–Q47P fusions.
**Figure S3** Complement to Figure 5: Relative amounts of mCystaTag–Q47P hybrids and free (released) cystatin domains in leaves expressing the fusions alone (−) or along with M2 (+).
**Figure S4** Complement to Figure 5: Intrinsic stability of mCystaTag–Q47P fusion hybrids in neutral to mildly acidic pH conditions.

**Table S1** Aliphatic index and instability index values calculated for the mCystaTag–Q47P fusions using the EXPASY server ProtParam tool.
