# Supplementary material for: Recombinant protein susceptibility to proteolysis in the plant cell secretory pathway is pH‐dependent
Source: Plant Biotechnol J. 2018 May 2;16(11):1928–38. doi: 10.1111/pbi.12928 (PMC6181212; doi:10.1111/pbi.12928)
Supplement: Supplementary file 1 — Figure S1 Intrinsic stability of GFP variant pHluorin in neutral to mildly acidic pH conditions. Figure S2 Complement to Figure 3: Structural models for the remaining 12 mCystaTag–Q47P fusions. Figure S3 Complement to Figure 5: Relative amounts of mCystaTag–Q47P hybrids and free (released) cystatin domains in leaves expressing the fusions alone (−) or along with M2 (+). Figure S4 Complement to Figure 5: Intrinsic stability of mCystaTag–Q47P fusion hybrids in neutral to mildly acidic pH conditions. [file PBI-16-1928-s001.pdf]

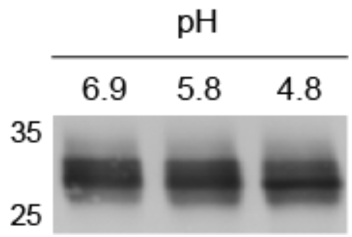


**Figure S1** Intrinsic stability of GFP variant pHluorin in neutral to mildly-acidic pH conditions. A protein extract prepared from pHluorin-expressing *N. benthamiana* leaf tissue was diluted 1:3 in 100 mM citric acid monohydrate/200 mM disodium phosphate buffer adjusted to pH 7, 6 or 5 to give final pH conditions of 6.9, 5.8 or 4.8, respectively. The resulting samples were incubated at 20˚C for 1 h, and then immunodetected with anti-GFP polyclonal antibodies following 12% (w/v) SDS-PAGE in reducing conditions and transfer on nitrocellulose sheets. Protein samples were supplemented with the cOmplete protease inhibitor cocktail (Roche Diagnostics) to prevent pHluorin proteolysis during incubation.

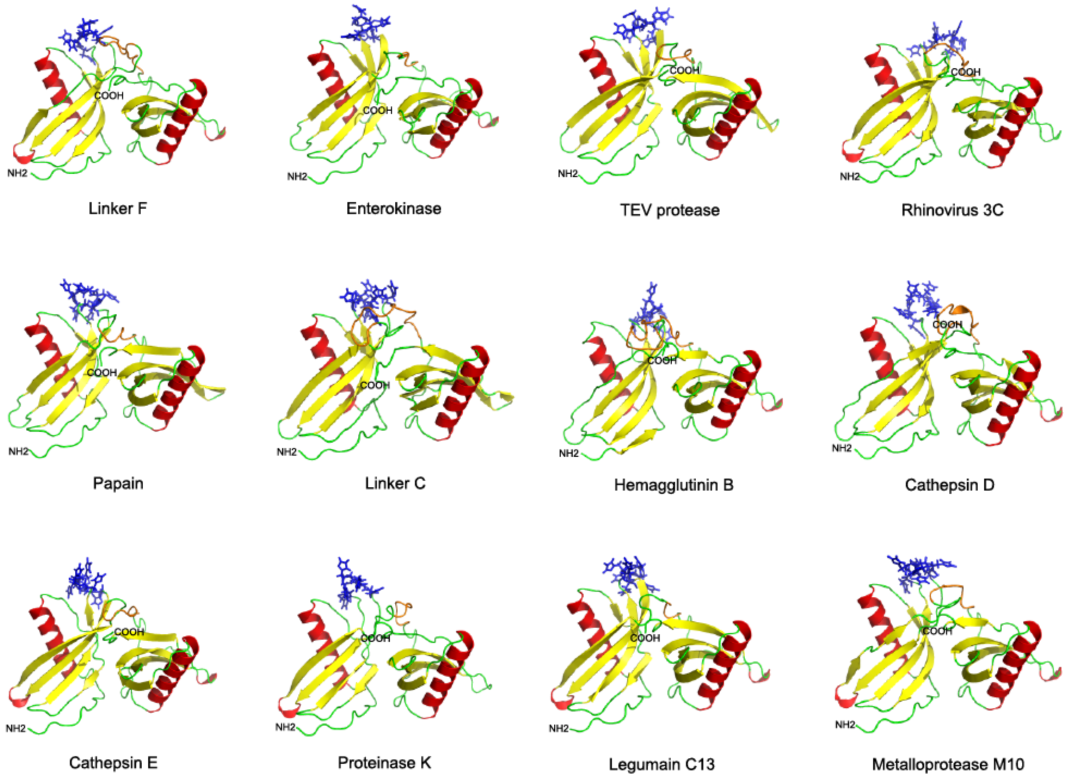


**Figure S2** Complement to Figure 3: Structural models for the remaining 12 mCystaTag–Q47P fusions. Models were built as for the fusions in Figure 2 using the NMR structure of rice cystatin I (PDB 1EQK) as a template. Peptide linkers are shown in orange, the poly-His motif of mCystaTag in blue. Ramachandran plots confirmed the stereochemical quality of these models (not shown).

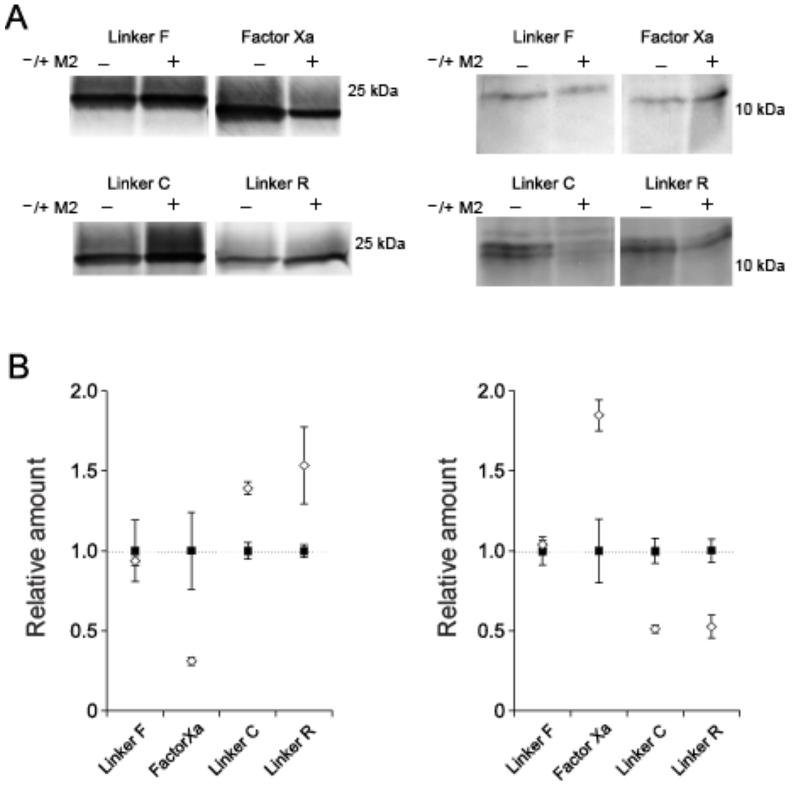


**Figure S3** Complement to Figure 5: Relative amounts of mCystaTag–Q47P hybrids and free (released) cystatin domains in leaves expressing the fusions alone (–) or along with M2 (+). (A) Immunodetection of mCystaTag–Q47P fusion hybrids (left) and free cystatin domains (right) in ELISA-assayed leaf extracts containing a fixed amount of antibody-reactive cystatin domains. Numbers on the right refer to molecular weight markers. (B) Relative amount of cystatin fusion (left) or free cystatins (right) for the hybrids expressed alone or along with M2, as determined by densitometric analysis of protein signals on non-saturated immunoblots. Data for the fusions co-expressed with M2 (white diamonds) are expressed relative to the signals in protein extracts of leaves transfected to express the corresponding fusion construct only (black squares; relative amount of 1.0). Data on panel B are the mean of three independent (plant) replicate values ± SE.

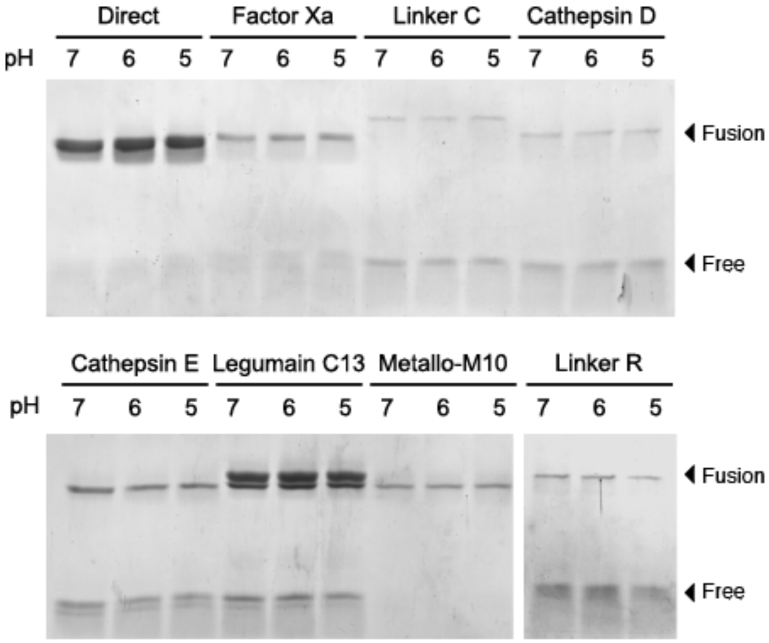


**Figure S4** Complement to Figure 5: Intrinsic stability of mCystaTag–Q47P fusion hybrids in neutral to mildly-acidic pH conditions. Cystatin fusions showing an increased or reduced amount in M2-transfected *N. benthamiana* leaves (Figure 5) were affinity-purified using the CystaTag-optimized IMAC purification protocol of Sainsbury *et al.* (2016),[1] and the eluted protein fractions filter-centrifuged to remove imidazole. Purified proteins were incubated at pH 5, 6 or 7 for 1 h at 20˚C, resolved by SDS-PAGE and stained with Coomassie blue. Similar amounts of protein were observed at the three pH for all tested fusions, except for Linker R fusion harbouring a rigid, acid-labile linker unstable in the plant cell secretory pathway (Jutras et al., 2015).[2]

[1] Sainsbury, F., Jutras, P. V., Vorster, J., Goulet, M.-C. and Michaud, D. (2016) A chimeric affinity tag for efficient expression and chromatographic purification of heterologous proteins from plants. *Front. Plant Sci.* **7**, 1–11.

[2] Jutras, P.V., D'Aoust, M.A., Couture, M.M.J.J., Vézina, L.P., Goulet, M.C., Michaud, D. and Sainsbury, F. (2015) Modulating secretory pathway pH by proton channel co-expression can increase recombinant protein stability in plants. *Biotechnol. J.* **10**, 1478–1486.
