# Supplementary material for: Recombinant protein susceptibility to proteolysis in the plant cell secretory pathway is pH‐dependent
Source: Plant Biotechnol J. 2018 May 2;16(11):1928–38. doi: 10.1111/pbi.12928 (PMC6181212; doi:10.1111/pbi.12928)
Supplement: Supplementary file 2 — Table S1 Aliphatic index and instability index values calculated for the mCystaTag–Q47P fusions using the EXPASY server ProtParam tool. [file PBI-16-1928-s002.pdf]

Table S1

Aliphatic index [1] and instability index [2] values calculated for the mCystaTag–Q47P fusions using the EXPASY server ProtParam tool (http://web.expasy.org/protparam)

	Aliphatic index	Instability index
Direct fusion	88,47	16,95
Linker F	83,37	20,11
Factor Xa	88,65	20,78
Enterokinase	86,27	16,35
TEV protease	87,34	16,71
Rhinovirus 3C protease	90,25	17,20
Thrombin	89,21	17,43
Papain	89,70	17,12
Linker C	82,57	23,26
Hemagglutinin B	89,25	14,78
Cathepsin D	92,74	15,73
Cathepsin E	87,89	18,56
Proteinase K	87,70	17,77
Metalloprotease M10	86,40	18,89
Linker R	86,45	16,45

[1] Ikai, A. (1980) Thermostability and aliphatic index of globular proteins. *J. Biochem.* **1898**, 1895–1898.

[2] Guruprasad, K., Reddy, B.V.B. and Pandit, M.W. (1990) Correlation between stability of a protein and its dipeptide composition: A novel approach for predicting *in vivo* stability of a protein from its primary sequence. *Prot. Eng. Des. Select.* **4**, 155–161.
